# Human parvovirus B19 nonstructural protein NS1 enhanced the expression of cleavage of 70 kDa U1-snRNP autoantigen

**DOI:** 10.1186/1423-0127-17-40

**Published:** 2010-05-25

**Authors:** Bor-Show Tzang, Der-Yuan Chen, Chun-Chou Tsai, Szu-Yi Chiang, Tsung-Ming Lin, Tsai-Ching Hsu

**Affiliations:** 1 Institute of Biochemistry and Biotechnology, Chung Shan Medical University, Taichung, Taiwan; 2 Department of Biochemistry, School of Medicine, Chung Shan Medical University, Taichung, Taiwan; 3 Clinical Laboratory, Chung Shan Medical University Hospital, Taichung, Taiwan; 4 Department of Allergy, Immunology and Rheumatology, Taichung Veterans General Hospital, Taichung, Taiwan; 5 Faculty of Medicine, National Yang-Ming University, Taipei, Taiwan; 6 School of Medicine, Chung-Shan Medical University and Institute of Biomedical Science, National Chung-Hsing University, Taichung, Taiwan; 7 Institute of Immunology, Chung Shan Medical University, Taichung, Taiwan; 8 Department of Health, Executive Yuan, Hua-Lien Hospital, Hua-Lien, Taiwan

## Abstract

**Background:**

Human parvovirus B19 (B19) is known to induce apoptosis that has been associated with a variety of autoimmune disorders. Although we have previously reported that B19 non-structural protein (NS1) induces mitochondrial-dependent apoptosis in COS-7 cells, the precise mechanism of B19-NS1 in developing autoimmunity is still obscure.

**Methods:**

To further examine the effect of B19-NS1 in presence of autoantigens, COS-7 cells were transfected with pEGFP, pEGFP-B19-NS1 and pEGFP-NS1K334E, a mutant form of B19-NS1, and detected the expressions of autoantigens by various autoantibodies against Sm, U1 small nuclear ribonucleoprotein (U1-snRNP), SSA/Ro, SSB/La, Scl-70, Jo-1, Ku, and centromere protein (CENP) A/B by using Immunoblotting.

**Results:**

Significantly increased apoptosis was detected in COS-7 cells transfected with pEGFP-B19-NS1 compared to those transfected with pEGFP. Meanwhile, the apoptotic 70 kDa U1-snRNP protein in COS-7 cells transfected with pEGFP-B19-NS1 is cleaved by caspase-3 and converted into a specific 40 kDa product, which were recognized by anti-U1-snRNP autoantibody. In contrast, significantly decreased apoptosis and cleaved 40 kDa product were observed in COS-7 cells transfected with pEGFP-NS1K334E compared to those transfected with pEGFP-B19-NS1.

**Conclusions:**

These findings suggested crucial association of B19-NS1 in development of autoimmunity by inducing apoptosis and specific cleavage of 70 kDa U1-snRNP.

## Background

Human parvovirus B19 (B19) has been associated with the development of various autoimmune disorders [1-7]. Evidences have indicated that many clinical features in patients with acute or chronic B19 infection are extremely similar to those with autoimmune diseases, including the elevated levels of autoantibodies [5,6,8-10]. However, the molecular basis and pathogenesis of B19-induced autoimmunity is still unclear.

B19 was firstly discovered in 1975 and known as a human pathogen [11]. The genome of B19 consists of three encoding regions including the nonstructural protein (NS1) and two capsid proteins, VP1 and VP2 [3,12]. B19-NS1 protein has been reported to act as a transactivator of the B19 viral p6 and various cellular promoters including tumor necrosis factor-α (TNF-α) or interleukin (IL)-6 [13-16]. Additionally, B19-NS1 is known to be involved in DNA replication, cell cycle arrest and the initiation of apoptosis in erythroid lineage and non-erythroid lineage cells [17-20]. Recently, many studies also implied the roles of B19-NS1 in development of autoimmunity that could be associated with B19-NS1 induced apoptosis [16,21,22]. However, no further investigation was performed or reported.

Apoptosis is known as a predominant cause for leakage of various autoantigens such as nucleosomal DNA, SSA/Ro, SSB/La, U1 small nuclear ribonucleoprotein (U1-snRNP) and phospholipid in patients with SLE or antiphospholipid syndrome (APS) [23-25]. The U1-snRNP complex is a common target for autoantibodies in serum of patients with SLE or mixed connective tissue disease (MCTD) [26,27]. Previous studies have demonstrated a specific cleavage of the 70-kDa protein component of the U1-snRNP by caspase 3 and caspase 9, which is recognized as a biochemical feature of apoptotic cell death [23,24,28]. The cleaved 70-kDa U1-snRNP will be converted into a C-terminally truncated 40-kDa protein fragment. Additionally, high recognition of the 40-kd apoptotic fragment of 70 kDa U1-snRNP has been shown to correlate with the presence of lupus-like skin disease in patients with anti-U1-70 kDa antibodies [29]. These findings indicated that apoptotically modified 70 kDa U1-snRNP is a candidate to drive anti-RNP reactivity in autoimmune disorders.

Previously, we had firstly reported the mitochondrial related apoptosis in B19 NS1-transfected epithelial COS-7 cells, which provides alternative information for B19-NS1 protein in B19 non-permissive cells [19]. In current study, we further investigated the effects of B19-NS1 in presence of autoantigens and found the increased specific cleaved product of 40 kDa U1-snRNP that was recognized by anti-RNP antibodies.

## Methods

### Patients and serum

Three volunteer in-patients from Division of Allergy, Immunology and Rheumatology, participated in this study, approved by Institutional Review Board (IRB), Taichung Veterans General Hospital, Taichung, Taiwan. All patients were infected with B19 virus and suffered from MCTD or SLE. The serum samples form the three patients contains IgG against B19 and RNP (ranges 170.5~181.4 Units). The serum samples from ten healthy individuals were used as controls. All healthy individuals and patients willing to volunteer were accepted without exclusion and diagnosis was made by a single board certified physician who is also our coauthor (Dr. Der-Yuan Chen).

### Plasmids and site-directed mutagenesis

Plasmid pEGFP-C1 was purchased from CLONTECH (CLONTECH Laboratories, Palo Alto, CA, USA). Plasmid pQE40-NS1, containing the NS1 gene of B19, was kindly provided by Professor Susanne Modrow, from the Institute for Medical Microbiology, Universität Regensburg, Regensburg, Germany. The NS1 open reading frame was obtained by PCR by using a 5'primer (5'-GCAGATCTATGGAGCTATTT AGAGGGGTG-3') and a 3' primer (5'-GCGTCGACCTCATAATCTACAAAGC TTTGC-3') containing *Bgl II *and *Sal I *recognition sequences for subsequent cloning. Amplified B19-NS1 DNA cDNA was ligated into the *Bgl II *and *Sal I *cloning site of the pEGFP-C1 vector (EGFP). The PCR was performed with reagents containing a 0.2 μM primers mixture, 1.25 μM dNTP mixture, 1.5 μM MgCl_2_, 10 ng template, and 2.5 U DNA polymerase (Takara, Tokyo, Japan). The pEGFP-NS1 was then transformed into DH5α competent cells, which were obtained from Life Technologies (Life Technologies, Carlsbad, California, USA). Restriction enzyme digestion, PCR and DNA sequencing analysis were used to verify the plasmid. A previous study has indicated that cytotoxic effect of B19-NS1 was abolished by single substitutions of amino acids within the NTP-binding domain [30]. To construct the mutant plasmid of B19-NS1, pEGFP-NS1-K334E, the QuikChange XL site-Directed Mutagenesis Kit (Stratagene, La Jolla, CA) was used to engineer a lysine to glutamic acid (K334E) mutation into the wild-type NS1 expression vector, pEGFP-NS1, with the following oligonucleotide primers: 5'-CCGCCAAGTACAGGAGAAACAAACT-3' (forward primer) and 5'-AGTTTGTTTCTCCTGTACTTGGCGG-3' (reverse primer). The PCR reaction was performed according to the manufactures' instructions. Briefly, the amplification was performed in a 50 μl reaction volume containing 10× reaction buffer, 50 ng of dsDNA template, 200 μM of dNTPs, 125 ng of each primer and 2.5 units of *PfuTurbo *DNA polymerase by using a Perkin-Elmer Gene Amp PCR system 2400. The PCR products were checked on a 1% agarose gel. Successful mutagenesis was verified by sequencing forwardly and reversely.

### Cell culture and Transfection

COS-7 cells were originally obtained from American type culture collection (ATCC) (Manassas, Va, USA) and grown in Dulbecco's modified Eagle medium (DMEM) supplemented with 10% fetal bovine serum (FBS) (GIBCO-BRL, Carlsbad, California, USA) at 37°C in a 5% CO_2 _incubator. A total of 1 × 10^6 ^cells were grown to 70% confluence in 100 mm^2 ^dishes before transfection. The transfection reaction was performed by using Lipofectamine plus reagents (Invitrogen, California, USA) with 2 μg of each plasmid, EGFP, EGFP-NS1 or EGFP-NS1K334E, according to the manufacturer's instructions. The cells were then cultured in serum-free DMEM for 12 hrs at 37°C in a 5% CO_2 _incubator and subsequently in DMEM with 10% FBS. The stable clones were obtained by G418 selection at the concentration of 800 mg/ml (Promega, Madison, Wisconsin, USA) in DMEM containing 10% FBS. For caspase-9 inhibition, the cells were pretreated for 12 h with 10 mM caspase-9 inhibitor (Z-LEHD-FMK) (Santa Cruz, CA, USA) and the cell lysate were then collected for Immunoblotting as described in our previous study [19].

### Fluorescence Microscopy

EGFP, EGFP-NS1 and EGFP-NS1K334E expression in transfected COS-7 cells were observed with a Zeiss Axioplan-2 epifluorescence microscope equipped with a fluorescence filter. Digital images of the cells were recorded by using a spot camera system.

### Flow cytometric analysis

The procedures for flow cytometric analysis were the same as used previously.^19 ^The cells (~2 × 10^6^) were fixed in 75% alcohol for 12-16 hr at 4°C, followed by RNase (1 mg/mL) treatment at 25°C for 30 min and analyzed by a flow cytometer (FACScan, Becton Dickinson, Bedford, MA, USA).

### RT-PCR

All studies were carried out in a designated PCR-clean area. RNA was extracted from infected cells using Trizol reagent (Invitrogene, Carlsbad, California, USA) according to the manufacture's instruction. Total RNA was isolated from EGFP, EGFP-NS1 and EGFP-NS1K334E expression cells. RNA samples were resuspended in diethyl pyrocarbonate (DEPC)-treated water, quantified, and then stored at -80°C until use. RNA concentration and purity were determined by a spectrophotometer by calculating the ratio of optical density at wavelengths of 260 and 280 nm. The first-strand cDNA for RT-PCR was synthesized from total RNA (2 μg) using the Promega RT-PCR system (Promega, Madison, Wisconsin, USA). The cDNAs encoding human B19 NS1 and GAPDH were amplified by RT-PCR using the following primer pairs: 5'-GGGGGGCCAGGGTTAAACCCCAGA -3' (forward primer for NS1 cDNAs, nt 694-717), 5'-CTTTAACACATGCTGCCCCACCAA-3' (reverse primer for NS1 cDNAs, nt1348-1371), 5'-CCATGGCACCGTCAAGGCTG A-3' (forward primer for GAPDH cDNAs), and 5'-TTGGCAGTGGGGACACGGA A-3' (reverse primer for GAPDH cDNAs). The amplification was performed in a 50 μl reaction volume containing 1× reaction buffer (Promega, Madison, Wisconsin, USA), 1.5 μM of MgCl_2_, 200 μM of dNTPs, 1 μM of each primer and 2.5 units of Taq DNA polymerase (Promega, Madison, Wisconsin, USA) using a Perkin-Elmer Gene Amp PCR system 2400. Each cycle consists of denaturation at 95°C for 1 min, annealing at 55°C for 45 sec, and amplification at 72°C for 45 sec. The RT-PCR derived DNA fragments obtained by 30 PCR cycles were subjected to electrophoresis on a 1.7% agarose gel.

### Caspase 3 activity assay

A caspase-3 ELISA kit (BD Pharmingen, San Diego, California, USA) was used for in vitro determination of caspase-3 activity in cell lysates according to manufacturer's instruction.

### Immunoblotting

The COS-7 cells transfected with different plasmids were lysed in an aliquot volume of 600 ul PRO-PREP^® ^solution (iNtRON Biotech, Korea) for 30 min on ice. The cell lysates were then centrifuged at 15,000 rpm for 10 min at 4°C and the supernatant was isolated and stored at -80°C until use. Protein concentration was determined according to the method described by Bradford [31]. Thirty μg of each protein sample were applied and separated onto a 12.5% sodium dodecyl sulfate-polyacrylamide gel electrophoresis (SDS-PAGE) [32] at 100-120 V for 1.5 hr before transferred to the nitrocellulose membranes [33]. The membranes were then cut into strips and soaked in 5% nonfat dry milk in PBS for 30 min at room temperature to saturate irrelevant protein binding sites. Antibodies against actin (Upstates, Charlottesville, Virginia, USA), Cleaved Caspase-3 (Asp175), Cleaved Caspase-9 (Asp315) (Cell Signaling, Massachusetts, USA) and Bax, Ku70/Ku-86 (Santa Cruz, CA, USA), SSA/Ro, SSB/La, Scl-70, Jo-1 (MBL, Nagoya, Japan), Sm, U1-snRNP, CENP A and B (INOVA Diagnostics, Inc San Diego, CA, USA), and B19-NS1 [16,19], were diluted in PBS with 2.5% BSA and incubated for 1.5 hr with gentle agitation at room temperature. The antigen-antibody was then hybridized with horseradish peroxidase (HRP) conjugated secondary antibody and detected with Pierce's Supersignal West Dura HRP Detection Kit (Pierce Biotechnology Inc., Rockford, IL). Quantified results were performed by densitometry (Appraise, Beckman-Coulter, Brea, California, USA).

### Statistical analysis

Statistical analysis was performed using the paired t test. Correlation between variables was assessed by Spearman's correlation coefficient and Pearson correlation. Regression parameter at 0.05 probability was considered significant.

## Results

### Expression of EGFP, EGFP-NS1 and EGFP-NS1K334E in COS-7 cells

**Figure 1:**
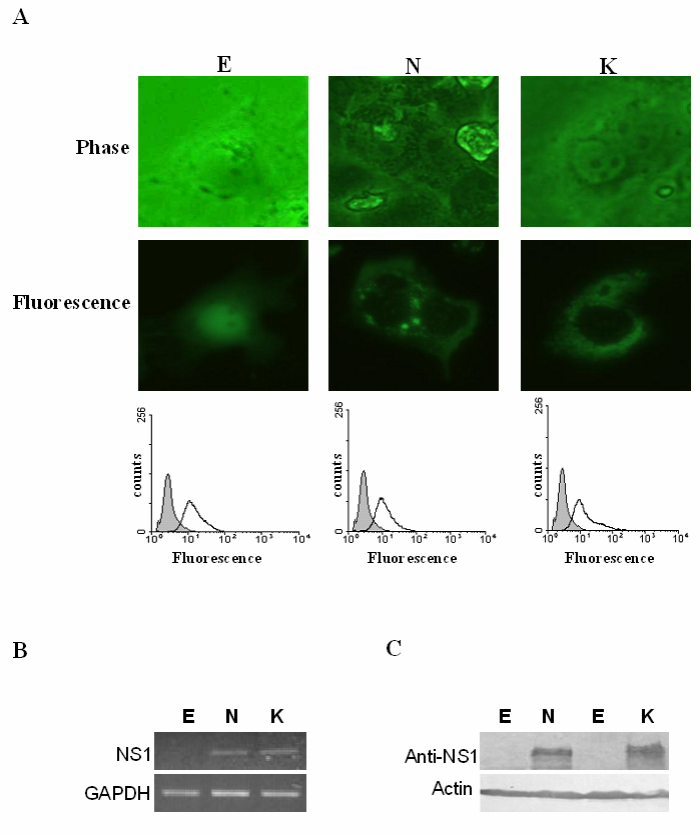
**Expression of EGFP, EGFP-NS1 and EGFP-NS1K334E**. (A) The COS-7 cells transfected with pEGFP, pEGFP-NS1 or pEGFP-NS1K334E were observed under phase contrast (upper panel) and fluorescence microscope (middle panel). The fluorescence intensity was also detected using flowcytometry (lower panel). The (B) mRNA and (C) protein expressions of B19-NS1 were detected by RT-PCR and Immunoblottings. E, N and K indicate EGFP, EGFP-NS1 and EGFP-NS1K334E, respectively.

Constructions of pEGFP, pEGFP-NS1, and pEGFP-NS1K334E and the stable clones of COS-7 cells were performed as described in materials and methods. The expression of EGFP, EGFP-NS1, and EGFP-NS1K334E in COS-7 cells were examined and confirmed with fluorescence microscope, flowcytometry, RT-PCR and immunoblots. The COS-7 cells transfected with pEGFP, pEGFP-NS1, or pEGFP-NS1K334E were observed under phase contrast and fluorescence microscope (Fig 1A). Markedly green fluorescence was observed in all transfectants (Fig 1A, middle panel) as well as the fluorescence shift detected by flowcytometry (Fig 1A, lower panel). To further confirm the expression of EGFP, EGFP-NS1, and EGFP-NS1K334E in COS-7 cells, RNA and protein expressions of NS1 were examined. Figure 1B revealed the RT-PCR results of B19-NS1. Expression of B19-NS1 was detected in COS-7 cells transfected with pEGFP-NS1 and pEGFP-NS1K334E but not pEGFP. Additionally, expressions of B19-NS1 protein were detected in COS-7 cells transfected with pEGFP-NS1 and pEGFP-NS1K334E but not pEGFP (Fig 1C).

### Presence of apoptosis in COS-7 cells transfected with pEGFP, pEGFP-NS1 and pEGFP-NS1K334E

**Figure 2:**
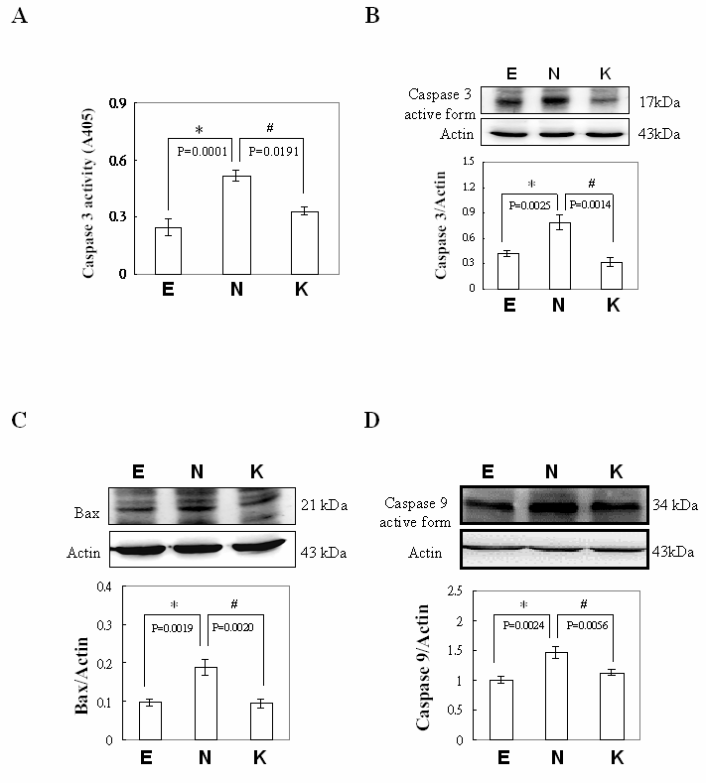
**Presence of apoptosis in COS-7 cells transfected with pEGFP, pEGFP-NS1 and pEGFP-NS1K334E**. (A) The activity of caspase-3, (B) presence of activated caspase-3, (C) Bax and (D) activated caspase-9 were detected by ELISA or Immunoblottings. E, N and K indicate EGFP, EGFP-NS1 and EGFP-NS1K334E, respectively. * and # indicate significant difference as compared to E or N , respectively.

To confirm the presence of apoptosis in COS-7 cells transfected with pEGFP, pEGFP-NS1, and pEGFP-NS1K334E, caspase-3 activity assay and immunoblottings were performed. Significantly higher activity of caspase-3 was detected in COS-7 cells transfected with pEGFP-NS1 compared to those transfected with pEGFP. In contrast, significantly reduced caspase-3 activity was observed in COS-7 cells transfected with pEGFP-NS1K334E compared to those transfected with pEGFP-NS1 (Fig 2A). Similar results were found in expressions of mitochondrial dependent apoptotic components including caspase-3, Bax and caspase-9 proteins. Significantly higher expressions of activated caspase-3, Bax, activated caspase-9 proteins were detected in COS-7 cells transfected with pEGFP-NS1 compared to those transfected with pEGFP whereas the significantly reduced levels of activated caspase 3, Bax, activated caspase-9 proteins were observed in COS-7 cells detected pEGFP-NS1K334E compared to those transfected with pEGFP-NS1 (Fig 2B, 2C and 2D).

### Cleavage apoptotic70-kDa U1-snRNP protein in COS-7 cells transfected with pEGFP-NS1

**Figure 3:**
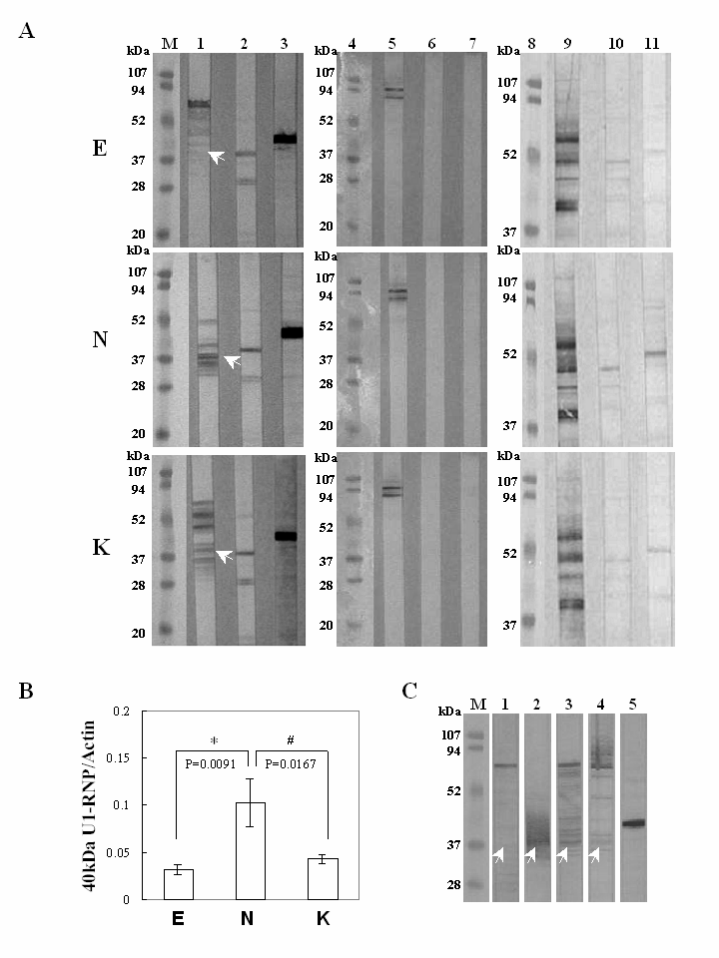
**Cleavage of 70 kDa U1-snRNP in COS-7 cells transfected with pEGFP-NS1**. (A) Expression of various autoantigens including 70 kDa U1-snRNP, Sm, Ku, CENPA/B, Scl-70, SSA/Ro, SSB/La and Jo-1 (lane 1-2, 5-7, 9-11) were detected in COS-7 cells transfected with pEGFP, pEGFP-NS1 and pEGFP-NS1K334E by various antibodies. Actin is used as control (lane 3) and arrow indicates a cleaved 40 kDa U1-snRNP protein. (B) Ratio of 40 kDa U1-snRNP to actin. (C) The presence of 70 kDa and 40 kDa U1-snRNP were detected in COS-7 cells transfected with (lanes 1 to 4) pEGFP, pEGFP-NS1, pEGFP-NS1K334E and pEGFP-NS1 with caspase-9 inhibitor by antibodies against U1-snRNP. (Lane 5) Actin is used as control. Three independent tests were performed. * and # indicate significant difference as compared to E or N, respectively. E, N and K indicate EGFP, EGFP-NS1 and EGFP-NS1K334E, respectively.

**Figure 4:**
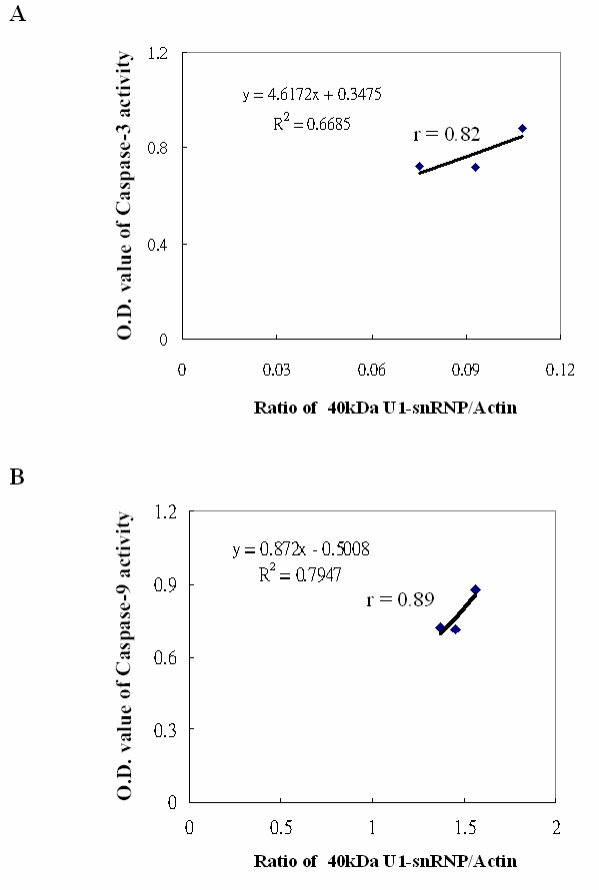
**Correlation of (A) caspase-3 activity and (B) caspases-9 to the 40 kDa U1-RNP in COS-7 cells transfected with pEGFP-NS1**.

**Figure 5:**
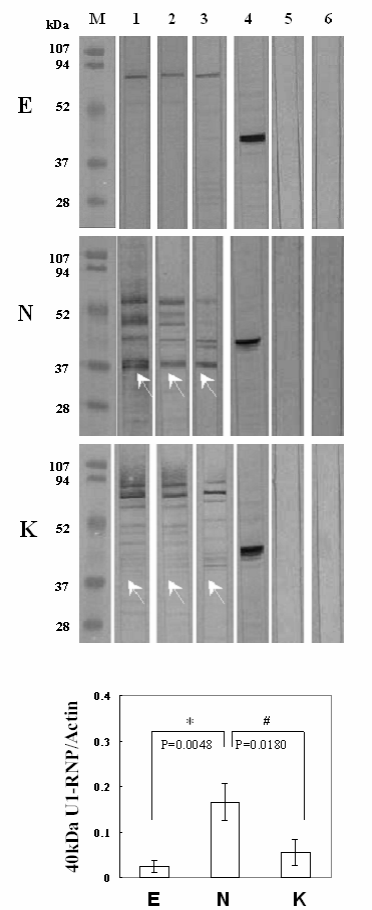
**Recognition of U1-snRNP in COS-7 cells by serum from patients with B19 infection**. (A) Presence of 70 kDa U1-snRNP and its cleaved 40 kDa-form in COS-7 cells transfected with pEGFP, pEGFP-NS1 and pEGFP-NS1K334E were detected with serum from B19 infected patients. Lanes 1 to 3 indicate serum from 3 different patients with B 19 infection. Actin is used as control (lane 4). Lanes 5 to 6 indicates representative results of serum samples that were randomly selected from the 10 healthy individuals. (B) Ratio of 40 kDa U1-snRNP to actin. Three independent tests were performed. * and # indicate significant difference as compared to E or N, respectively. E, N and K indicate EGFP, EGFP-NS1 and EGFP-NS1K334E, respectively.

Since B19-NS1 has been known to induce apoptosis and linked to the pathogenesis of autoimmune disorders, herein we further examined the presence of various autoantigens, including Sm, U1-snRNP, SSA, SSB, Scl-70, Jo-1, Ku, CENP-A/B. The presence of Sm, U1-snRNP, Ku, SSA, SSB and Jo-1 was detected in COS-7 cells transfected with pEGFP, pEGFP-NS1 and pEGFP-NS1K334E, whereas no expression of Scl-70, and CENP-A/B proteins were detected (Fig 3). Interestingly, a 40 kDa protein that was recognized by antibody against 70 kDa U1-snRNP was markedly increased in COS-7 cells transfected with pEGFP-NS1 (Fig 3, middle panel). In contrast, less presence of 40 kDa protein was observed in COS-7 cells transfected with pEGFP-NS1k334E (Fig 3, lower panel and 3B). Additionally, the 40 kDa U1-snRNP in COS-7 cells transfected with pEGFP-NS1 was markedly reduced in the presence of caspase-9 inhibitor (Fig 3C). Meanwhile, significant correlation was found between caspase-3 activity and 40 kDa U1-snRNP/actin ratio in COS-7 cells transfected with pEGFP-NS1 (Fig 4A, r = 0.82). Meanwhile, caspases-9 activity was also significantly correlated to the 40 kDa U1-snRNP/actin ratio in COS-7 cells transfected with pEGFP-NS1 (Fig 4B, r = 0.89). To further verify the association among the cleaved 70 kDa U1-snRNP, B 19 infection and autoimmunity, serum samples from 3 different B 19 infected patients with MCTD or SLE were used to detect the U1-snRNP in COS-7 cells transfected with pEGFP, pEGFP-NS1 and pEGFP-NS1K334E. The presence of 40 kDa U1-snRNP was recognized by all serum samples in pEGFP-NS1 transfected COS-7 cells (Fig 5).

## Discussion

Human parvovirus B 19-NS1 has been known to induce apoptosis that may contribute to the development of autoimmunity. However, no further evidence about the mechanism was reported. In current study, we reported the significantly increased cleaved 40 kDa-U1-RNP in COS-7 cells transfected with pEGFP-NS1 compared to those transfected with pEGFP. In contrast, significantly reduced 40 kDa-U1-RNP product was observed in COS-7 cells transfected with pEGFP-NS1K334E as well as the apparently reduced caspase-3 activity and apoptosis.

Apoptosis is a leading cause of autoantigens, which have been increasingly recognized as crucial targets of autoantibodies across a broad spectrum of autoimmune disorders such as SLE and antiphospholipid syndrome (APS) [23-25]. Previously, B 19-NS1 has been reported to play crucial roles in cell cycle arrest and apoptosis in both erythroid lineage cells [17,18] and non-erythroid lineage cells [19,20]. A recent study also reported that over-expressed B 19-NS1 triggers activation of caspase-3 and degradation of Na+/H+ exchanger, which may contribute to apoptosis and thus participate in the pathological process [34]. Besides, the cytotoxic effect of B 19-NS1 can be abolished by a single substitution of amino acids within the NTP-binding domain [30] and has been directly related to the induction of apoptosis [35,36], which was used as a negative control of B 19-NS1. Although these findings have demonstrated the association between B19-NS1 and apoptosis, however, no evidence is provided about the effect of mutated B19-NS1 on apoptosis. In this study, we revealed the significantly increased apoptosis in COS-7 cells transfected with pEGFP-NS1 compared to those transfected with pEGFP. In contrast, significantly reduced apoptosis was observed in COS-7 cells transfected with pEGFP-NS1K334E compared to those transfected with pEGFP-NS1. These data suggest that B19-NS1 induced apoptosis is probably associated with the cytotoxic effect of B19-NS1. However, further investigation is still needed to verify the details.

Growing evidences suggest that deregulation of apoptosis is involved in development of autoimmunity. Indeed, various apoptotic cell antigens, including fragmented endoplasmic reticulum, ribosomes, ribonucleoprotein, nucleosomal DNA, SSA/Ro, SSB/La, and small nuclear ribonucleoproteins, have been recognized as targets of autoantibodies across a broad spectrum of autoimmune diseases [23-25,37]. Moreover, various autoantigenic proteins are cleaved during apoptosis by caspases, a family of cysteinyl asparate-specific proteinases [37,38]. The specific 70 kDa U1-snRNP protein is known as an autoantigenic protein for autoantibodies in serum from the patients with SLE or MCTD [26,27]. The specific 70 kDa U1-snRNP, cleaved during apoptosis by caspase 3 [23,24] and caspase 9 [28] and converted it into a C-terminally truncated 40 kDa protein fragment. High recognition of the 40-kd apoptotic fragment of U1-70 kDa has been shown to correlate with the presence of lupus-like skin disease in patients with antibodies against 70 kDa U1-snRNP [29,39,40]. Thus, apoptotically modified 70 kDa U1-snRNP antigen is considered as a candidate to drive anti-RNP autoimmunity in lupus. As shown in current study, significant increase of cleaved 40 kDa fragment from the specific 70 kDa U1-snRNP protein was observed in COS-7 cells transfected with pEGFP-NS1 but not in those transfected with pEGFP-NS1K334E, which has high correlations with increased caspase-3 and caspase-9 activities. Since the serum from B19 infected patients with MCTD or SLE recognized the B19-NS1-induced cleavage of 70 kDa U1-snRNP, however, it could suggest the association between B19-NS1 induced cleavage of 70 kDa U1-snRNP by B19-NS1 and autoimmunity. Although the precise role of NS1 in inducing autoimmunity is still obscure, here we firstly report the increased cleaved 40 kDa RNP is associated with B19-NS1 and recognized by B19 infected patients. that has been recognized as an indicator for development of autoimmunity.

## Conclusion

Altogether, we have indicated that B19-NS1 induced apoptosis is probably associated with the cytotoxic effect of B19-NS1. Additionally, the B19-NS1 induced apoptosis leads to a specific cleavage of 70 kDa U1-snRNP and is suggested to play crucial roles in development of autoimmunity.

## Competing interests

The authors declare that they have no competing interests.

## Authors' contributions

BST conceived this study, drafted the manuscript, and performed the statistical analyses. CCT performed the Immunofluoresence, RT-PCR and Immunoblotting. DYC and SYC provided material support and encouragement for this work. TML performed the Immunoblotting. TCH provided material support and direction, drafted significant portions of the manuscript, and performed the Immunofluoresence, RT-PCR, flow cytometric analysis and Immunoblotting. All authors read and approved the final manuscript.

## References

[B1] Lehmann HW von Landenberg P Modrow S Parvovirus B19 infection and autoimmune disease Autoimmun Rev 2003 2 218 23 10.1016/S1568-9972(03)00014-4 12848949

[B2] Meyer O Parvovirus B19 and autoimmune diseases Joint Bone Spine 2003 70 6 11 10.1016/S1297-319X(02)00004-0 12639611

[B3] Young NS Brown KE Mechanisms of disease: Parvovirus B19 N Engl J Med 2004 350 586 597 10.1056/NEJMra030840 14762186

[B4] Servey JT Reamy BV Hodge J Clinical presentations of parvovirus B19 infection Am Fam Physician 2007 75 373 376 17304869

[B5] Tzang BS Tsay GJ Lee YJ Li C Sun YS Hsu TC The association of VP1 unique region protein in acute parvovirus B19 infection and antiphospholipid antibody production Clin Chim Acta 2007 378 59 65 10.1016/j.cca.2006.10.016 17169353

[B6] Tzang BS Lee YJ Yang TP Tsay GJ Shi JY Tsai CC Hsu TC Induction of antiphospholipid antibodies and antiphospholipid syndrome-like autoimmunity in naive mice with antibody against human Parvovirus B19 VP1 unique region protein Clin Chim Acta 2007 382 31 36 10.1016/j.cca.2007.03.014 17451664

[B7] Lunardi C Tinazzi E Bason C Dolcino M Corrocher R Puccetti A Human parvovirus B19 infection and autoimmunity Autoimmun Rev 2008 8 116 120 10.1016/j.autrev.2008.07.005 18700174

[B8] Chou CTN Hsu TC Chen RM Lin LI Tsay GJ Parvovirus B19 infection associated with the production of anti-neutrophil cytoplasmic antibody (ANCA) and anticardiolipin antibody (aCL) Lupus 2000 9 551 554 10.1177/096120330000900714 11035424

[B9] Von Landenberg P Lehmann HW Knoll A Dorsch S Modrow S Antiphospholipid antibodies in pediatric and adult patients with rheumatic disease are associated with parvovirus B19 infection Arthritis Rheum 2003 48 1939 1947 10.1002/art.11038 12847688

[B10] von Landenberg P Lehmann HW Modrow S Human parvovirus B19 infection and antiphospholipid antibodies Autoimmun Rev 2007 6 278 285 10.1016/j.autrev.2006.09.006 17412298

[B11] Cossart YE Field AM Cant B Widdows D Parvovirus-like particles in human sera Lancet 1975 1 72 3 10.1016/S0140-6736(75)91074-0 46024

[B12] Corcoran A Doyle S Advances in the biology, diagnosis and host-pathogen interactions of parvovirus B19 J Med Microbiol 2004 53 459 75 10.1099/jmm.0.05485-0 15150324

[B13] Fu Y Ishii KK Munakata Y Saitoh T Kaku M Sasaki T Regulation of tumor necrosis factor alpha promoter by human parvovirus B19 NS1 through activation of AP-1 and AP-2 J Virol 2002 76 5395 403 10.1128/JVI.76.11.5395-5403.2002 11991968 PMC137035

[B14] Mitchell LA Parvovirus B19 nonstructural (NS1) protein as a transactivator of interleukin-6 synthesis: common pathway in inflammatory sequelae of human parvovirus infections? J Med Virol 2002 67 267 74 10.1002/jmv.2217 11992589

[B15] Raab U Beckenlehner K Lowin T Niller HH Doyle S Modrow S NS1 protein of parvovirus B19 interacts directly with DNA sequences of the p6 promoter and with the cellular transcription factors Sp1/Sp3 Virology 2002 293 86 93 10.1006/viro.2001.1285 11853402

[B16] Hsu TC Tzang BS Huang CN Lee YJ Liu GY Chen MC Tsay GJ Increased expression and secretion of interleukin-6 in human parvovirus B19 non-structural protein (NS1) transfected COS-7 epithelial cells Clin Exp Immunol 2006 144 152 7 10.1111/j.1365-2249.2006.03023.x 16542377 PMC1809635

[B17] Morita E Sugamura K Human parvovirus B19-induced cell cycle arrest and apoptosis Springer Semin Immunopathol 2002 24 187 199 10.1007/s00281-002-0099-6 12503064

[B18] Morita E Nakashima A Asao H Sato H Sugamura K Human parvovirus B19 nonstructural protein (NS1) induces cell cycle arrest at G(1) phase J Virol 2003 77 2915 2921 10.1128/JVI.77.5.2915-2921.2003 12584315 PMC149759

[B19] Hsu TC Wu WJ Chen MC Tsay GJ Human parvovirus B19 non-structural protein (NS1) induces apoptosis through mitochondria cell death pathway in COS-7 cells Scand J Infect Dis 2004 36 570 7 10.1080/00365540410016230 15370668

[B20] Poole BD Karetnyi YV Naides SJ Parvovirus B19-induced apoptosis of hepatocytes J Virol 2004 78 7775 7783 10.1128/JVI.78.14.7775-7783.2004 15220451 PMC434113

[B21] Tsay GJ Zouali M Unscrambling the role of human parvovirus B19 signaling in systemic autoimmunity Biochem Pharmacol 2006 72 1453 1459 10.1016/j.bcp.2006.04.023 16764828

[B22] Lehmann HW Lutterbüse N Plentz A Akkurt I Albers N Hauffa BP Hiort O Schoenau E Modrow S Association of parvovirus B19 infection and Hashimoto's thyroiditis in children Viral Immunol 2008 21 379 383 10.1089/vim.2008.0001 18788945

[B23] Casciola-Rosen LA Anhalt G Rosen A Autoantigens targeted in systemic lupus erythematosus are clustered in two populations of surface structures on apoptotic keratinocytes J Exp Med 1994 179 1317 30 10.1084/jem.179.4.1317 7511686 PMC2191465

[B24] Casciola-Rosen LA Miller DK Anhalt GJ Rosen A Specific cleavage of the 70-kDa protein component of the U1 small nuclear ribonucleoprotein is a characteristic biochemical feature of apoptotic cell death J Biol Chem 1994 269 30757 30760 7983001

[B25] Levine JS Koh JS Subang R Rauch J Apoptotic cells as immunogen and antigen in the antiphospholipid syndrome Exp Mol Pathol 1999 66 82 98 10.1006/exmp.1999.2243 10331968

[B26] Klein Gunnewiek JMT Putte LBA van de van Venrooij WJ The U1-70 kDa snRNP complex: an autoantigen in connective tissue diseases Clin Exp Rheumtol 1997 15 549 560 9307865

[B27] Hof D Cheung K de Rooij DJ Hoogen FH van den Pruijn GJ van Venrooij WJ Raats JM Autoantibodies specific for apoptotic U1-70K are superior serological markers for mixed connective tissue disease Arthritis Res Ther 2005 7 R302 309 10.1186/ar1490 15743477 PMC1065328

[B28] Ramírez-Sandoval R Sánchez-Rodríguez SH Herrera-van Oostdam D Avalos-Díaz E Herrera-Esparza R Antinuclear antibodies recognize cellular autoantigens driven by apoptosis Joint Bone Spine 2003 70 187 194 10.1016/S1297-319X(03)00019-8 12814761

[B29] Greidinger EL Casciola-Rosen L Morris SM Hoffman RW Rosen A Autoantibody recognition of distinctly modified forms of the U1-70-kd antigen is associated with different clinical disease manifestations Arthritis Rheum 2000 43 881 8 10.1002/1529-0131(200004)43:4<881::AID-ANR20>3.0.CO;2-G 10765934

[B30] Momoeda M Wong S Kawase M Young NS Kajigaya S A putative nucleoside triphosphate-binding domain in the non-structural protein of B19 parvovirus is required for cytotoxicity J Virol 1994 68 8443 8446 7966641 10.1128/jvi.68.12.8443-8446.1994 PMC237320

[B31] Bradford MM A rapid and sensitive method for the quantitation of microgram quantities of protein utilizing the principle of protein-dye binding Anal Biochem 1976 72 248 254 10.1016/0003-2697(76)90527-3 942051

[B32] Laemmli UK Cleavage of structural proteins during the assembly of the head of bacteriophage T4 Nature 1970 227 680 685 10.1038/227680a0 5432063

[B33] Towbin H Staehelin T Gordon J Electrophoretic transfer of proteins from polyacrylamide gels to nitrocellulose sheets: procedure and some applications Proc Natl Acad Sci USA 1979 76 4350 4354 10.1073/pnas.76.9.4350 388439 PMC411572

[B34] Lupescu A Geiger C Zahir N Aberle S Lang PA Kramer S Wesselborg S Kandolf R Foller M Lang F Bock CT Inhibition of Na+/H+ exchanger activity by parvovirus B19 protein NS1 Cell Physiol Biochem 2009 23 211 220 10.1159/000204110 19255516

[B35] Utz PJ Anderson P Posttranslational protein modifications, apoptosis, and the bypass to tolerance to antigens Arthritis Rheum 1998 41 1152 1160 10.1002/1529-0131(199807)41:7<1152::AID-ART3>3.0.CO;2-L 9663470

[B36] Ozawa K Ayub J Kajigaya S Shimada T Young N The gene encoding the nonstructural protein of B19 (human) parvovirus may be lethal in transfected cells J Virol 1988 62 2884 2889 2969055 10.1128/jvi.62.8.2884-2889.1988 PMC253725

[B37] Nakashima A Morita E Saito S Sugamura K Human Parvovirus B19 nonstructural protein transactivates the p21/WAF1 through Sp1 Virology 2004 329 493 504 15518826 10.1016/j.virol.2004.09.008

[B38] Graham KL Utz PJ Sources of autoantigens in systemic lupus erythematosus Curr Opin Rheumatol 2005 17 513 517 10.1097/01.bor.0000171215.87993.6b 16093826

[B39] Greidinger EL Foecking MF Ranatunga S Hoffman RW Apoptotic U1-70 kd is antigenically distinct from the intact form of the U1-70-kd molecule Arthritis Rheum 2002 46 1264 1269 10.1002/art.10211 12115232

[B40] Greidinger EL Zang Y Jaimes K Hogenmiller S Nassiri M Bejarano P Barber GN Hoffman RW A murine model of mixed connective tissue disease induced with U1 small nuclear RNP autoantigen Arthritis Rheum 2006 54 661 669 10.1002/art.21566 16453294

